# Beyond disease-progression: Clinical outcomes after *EGFR*-TKIs in a cohort of *EGFR* mutated NSCLC patients

**DOI:** 10.1371/journal.pone.0181867

**Published:** 2017-08-04

**Authors:** Roxana Alina Tudor, Adrijana D'Silva, Alain Tremblay, Paul MacEachern, Don Morris, Darren Brenner, Karen Kopciuk, Dafydd Gwyn Bebb

**Affiliations:** 1 Department of Medical Oncology, Cumming School of Medicine, University of Calgary, Calgary-Alberta, Canada; 2 Division of Respiratory Medicine, Cumming School of Medicine, University of Calgary, Calgary-Alberta, Canada; 3 Arnie Charbonneau Cancer Institute, University of Calgary, Calgary-Alberta, Canada; 4 Cancer Epidemiology and Prevention Research, CancerControl Alberta, Alberta Health Services, Calgary-Alberta, Canada; 5 Department of Mathematics and Statistics, Faculty of Science, University of Calgary, Calgary-Alberta, Canada; Seconda Universita degli Studi di Napoli, ITALY

## Abstract

**Purpose:**

Treatment and clinical-outcomes were described in a sub-cohort of non-small-cell lung cancer (NSCLC) patients with disease-progression (PD) after epidermal growth factor tyrosine kinase inhibitors (*EGFR*-TKIs) treatment.

**Patients and methods:**

We retrospectively analyzed a single-institutional *EGFR* mutation positive (*EGFR*mut^+^) NSCLC cohort for post-TKI-PD management, and assessed overall survival (OS) and post-progression survival (PPS). All de-novo (first lung-cancer occurrence) stage IIIA-IV patients, as well as de-novo stage IV subset was analyzed. Multi-state modeling (MSM) and a Cox PH regression model with propensity score weights adjusted for clinicopathological variables between: diagnosis and PD and PD to death.

**Results:**

123 stage IIIA-IV patients were identified with 104 meeting RECIST-1.1-PD criteria. This RECIST-1.1-PD criteria subset included females (64.6%), Asians (39.4%), never/non-smokers (55.8%), and exon 19 deletion carriers (44.2%). Commonest treatment beyond initial-PD was continuing TKI alone (46/104), with another 21 patients continuing TKI plus additional systemic therapy. The median OS for patients who continued TKI treatment at initial-PD was 21.1 months versus 15.6 months for patients who discontinued TKI, *p* = 0.006. Via MSM analysis, continuing TKI at initial-PD followed by other systemic therapy was associated with an 83% reduced death risk, adjusted HR: 0.17 (95% CI: 0.07, 0.39). In the Cox PH model, ever-smokers with an exon 19 deletion had increased risk of death after PD (adjusted HR: 3.19, 95% CI: 1.54, 6.58), as did exon 21 mutation carriers, (adjusted HR: 2.10, 95% CI: 1.10, 4.00) and females (adjusted HR: 3.19, 95% CI: 1.54, 6.58).

**Conclusion:**

Subsequent systemic therapy after continuing TKI at initial-PD reduced the risk of death. Additionally, our data suggest that positive smoking history increases death risk for some *EGFR* mutation types and females.

## Introduction

Lung-cancer is a significant clinical-burden worldwide; each year, more than 1.6 million individuals are diagnosed and 1.4 million die from this disease, making it the leading cause of cancer-related mortality worldwide [1,2]. By 2030, the lung-cancer incidence is expected to increase to 2.2 million new cases per year [3].

Non-small cell lung cancer (NSCLC) accounts for approximately 85% of lung-cancer cases. Epidermal growth factor receptor (*EGFR*) mutations have been identified in ~ 30% of East-Asian patients with advanced-NSCLC and in 10–15% of patients in Western countries [4]. Treatment with *EGFR*-tyrosine kinase inhibitors (TKIs) in NSCLC patients harboring sensitizing *EGFR* mutations, is associated with significant survival benefits and better quality of life compared with conventional chemotherapy [5,6,8,9]. Particularly, *EGFR*-TKIs interfere with the function of the ATP-binding pocket, thereby preventing the phosphorylation and activation that would allow for cancer-cell survival and cell division promotion ^2^. Several clinical trials have shown that *EGFR*-TKIs (gefitinib, erlotinib, afatinib) produce higher response rates, longer progression-free survival, and are less toxic than platinum-based chemotherapy amongst *EGFR*mut^+^ NSCLC patients [5–9]. Many of these trials however, have been carried out in Asian- enriched populations, with notable exceptions: FIELT, EURTAC and IFUM trials, which included non-Asian patients [10,11,12].

Unfortunately, all *EGFR*mut^+^ patients treated with TKIs eventually experience disease-progression and ultimately die [2]. Various TKI-acquired resistance mechanisms have been studied, with the most common being the missense mutation within exon 20—T790M, found in approximately half of *EGFR*mut^+^ NSCLC patients treated with a TKI that have progressed [13]. In 2008, it was demonstrated that the T790M mutation changes the relative affinity of the EGF receptor in favor of competitive ATP binding within the ATP-binding pocket, thereby creating resistance to these drugs [14]. Although T790M has provided the clinical need for the development of third-generation TKIs, there are other intrinsic mechanisms of TKI-acquired resistance reported [13,15].

Guidelines addressing treatment after progression on a TKI vary depending on the PD-pattern and the presence of the commonest resistance mutation, T790M [16]. Recent data supports using third-generation *EGFR*-TKIs, such as osimertinib, when the T790M resistance mutation is detected [17]. However, during the timeframe of this retrospective analysis, osimertinib was not approved and guidelines recommended a switch to platinum-based chemotherapy. Historically, patients with systemic-PD are also more likely to be switched to platinum-based chemotherapy [18,19].

Although guidelines outlining subsequent *EGFR*-TKI-PD treatment exist [19,20], there is a common perception that patients continue treatment with a TKI often beyond initial-PD. Currently, there is limited evidence outlining the clinical benefits of continuing an *EGFR*-TKI treatment at initial-PD. In particular, the ASPIRATION and IMPRESS trials addressed the role of continuing *EGFR-*TKIs at the time of clinical progression; however, concluding data are debatable. In particular, the ASPIRATION trial highlighted that continuing a TKI leads to a benefit of 3.1 months, meanwhile the IMPRESS trial suggested a switch to chemotherapy after *EGFR*-TKI-PD. As a result, platinum-based doublet chemotherapy beyond PD remains the suggested standard of care, even for *EGFR*mut^+^ NSCLC patients [21]. Furthermore, with the advent of cell-free DNA testing upon progression and the results of the recently presented AURA 3 trial of osimertinib in this setting [22], there is little controversy that in 2016, osimertinib should be used in the setting of a T790M^+^ mutational progression.

With the aim of determining the impact of subsequent treatments on overall survival (OS) and post-progression survival (PPS), we performed a single-centre retrospective study in a sub-cohort of *EGFR*mut^+^ NSCLC patients with PD, post-TKI treatment.

## Materials and methods

### Study population

This retrospective study evaluated NSCLC patients diagnosed between March 16, 2010 and December 31, 2014 at the Tom Baker Cancer Centre (Calgary, Alberta). Specifically, 123 patients were characterized as ‘de-novo’ for a first lung-cancer occurrence. The inclusion criteria were: age > 18 years, histologically/-or cytologically confirmed stage IIIA, IIIB or IV, presence of *EGFR* mutations, and treatment with *EGFR*-TKIs (gefitinib, erlotinib). Patients were excluded if they previously received any radical therapy for an early-stage lung cancer (IA-IIB) diagnosis, and later relapsed with disease. Data were collected through the: Iressa^®^ Alliance program, Alberta Cancer Registry, Calgary Laboratory Services, the Glans-Look lung-cancer database (GLD) and the institutional electronic medical record (S1 Fig). This study was approved by the institutional review board of the University of Calgary (Conjoint Health Research Ethics Board—REB15-1189).

The time-period of the study represents the adaptation of routine *EGFR* mutation status analysis in Alberta in 2010. Further, only de-novo stage IIIA-IV *EGFR*mut^+^ NSCLC patients were included in this study to: (i) further reduce survival-bias and (ii) outline the early clinical-practice for *EGFR*mut^+^ patients with advanced disease.

The following baseline characteristics were collected: age, sex, ethnicity (Asian/or non-Asian), family lung-cancer history and smoking history. Ethnicity was defined according to country of birth. If a ‘suspected Asian’ ethnicity was identified through surname analysis, the patient was categorized as Asian. Smoking history was defined as: ‘never/non-smokers’ as patients who had never smoked or smoked fewer than 100 cigarettes in their lifetime, and ‘ever-smokers’ as patients who had a smoking history of more than 100 cigarettes in their lifetime and were still active smokers at the time of diagnosis, as well as patients who quit smoking cigarettes ≥ 3 months from time of diagnosis.

Tumor-related features included the clinical or pathological stage of the disease according to the tumor, node and metastasis (TNM) classification of the 7^th^ edition of the American Joint Committee on Cancer (AJCC) criteria [23]. Further, the type of *EGFR* mutation was collected. The ‘all other’ *EGFR* mutation category included: double mutations (exon 19 deletions and L858R, exon 19 deletions and L861Q, G719X and S768I, L861Q and G719X, T790M and L858R); as well as the less common single-*EGFR* mutations: G719X, L861Q, S768I; meanwhile others remained “unspecified” in the pathology report and/-or electronic medical record/dictations.

### Evaluating response to *EGFR*-TKI treatment

To evaluate tumor response, patients underwent chest radiography approximately every 2–4 weeks and chest computed tomography every 2–3 months at the discretion of the attending clinician. The disease status was assessed according to the Response Evaluation Criteria in Solid Tumors guideline, version 1.1 (RECIST-1.1) [24]. OS was measured from the date of diagnosis to date of death or the date of last follow-up visit for patients that were still alive. PPS was defined from date of initial disease-progression to death or date of last follow-up visit for patients who were still alive.

### Statistical analyses

The OS and PPS analyses were performed using the Kaplan-Meier method and the global log-rank test. In addition, the OS for stage IIIA-IV patients (N = 123) was assessed via a Markov multi-state modeling (MSM) regression approach (*mstate* R package), meanwhile PPS was carried out via a Cox proportional hazards (PH) regression model with propensity score weights for stage IV patients (N = 94; S2 Fig). The average treatment effect (ATE) propensity score weights were calculated using logistic regression that modeled patient, tumour and treatment predictors from diagnosis, as well as the progression-free duration on the decision to continue or discontinue TKI at initial-PD (*twang* R package) [25]. Descriptive analyses included categorical data summarized by frequencies and percentages, meanwhile continuous covariates were indicated with a median, and the first and third quartiles, Q_1_ and Q_3,_ respectively. Statistical significance was considered at a level of α = 0.05.

All pairwise interactions between the predictors of interest (gender, smoking history, ethnicity and *EGFR* mutation type) were evaluated in both regression models for the time since PD to death or last follow-up date. Further, they were removed based on non-significant likelihood ratio tests, followed by the predictor, if it was not present in any interactions. The PH assumption was evaluated for all predictors in the MSM and Cox PH regression models by testing for non-zero slopes between the scaled Schoenfeld residuals and log(time). Index plots of *dfbetas* for predictors in the Cox PH regression analysis were also carried out (S3 Fig); no influential values were found. The propensity score model was evaluated for sufficient number of trees, level of interactions and balance—the latter through an effect-size plot showing the reduction in the magnitude of the group differences of the clinicopathological variables, and with a Q-Q plot showing *t*-test *P*-values with and without weighting (S4 Fig).

## Results

### Patient characteristics

123 patients with stage IIIA, IIIB or IV disease were identified with, (i) 104 who developed PD according to RECIST-1.1, and (ii) 94 out of the 104 sub-cohort, were patients with stage IV disease only, with met RECIST-1.1-PD. Table 1 summarizes patient, tumor and treatment characteristics.

**Table 1:** Baseline clinicopathological features of *EGFR*mut^+^ NSCLC patients (N = 104/123; N = 94/104).

	*EGFR*mut^+^ NSCLC IIIA-IV with RECIST-1.1-PD	*EGFR*mut^+^ NSCLC stage IV only with RECIST-1.1-PD
**Clinicopathological feature**	**N = 104/123[Q**_**1**_**, Q**_**3**_**]**	**N = 94/104[Q**_**1**_**, Q**_**3**_**]**
**Median age, *years***	65.6*yrs* [54.2*yrs*, 74.05*yrs*]	65.05*yrs* [54.0*yrs*, 74.3*yrs*]
**Median overall survival, *months***	19.2*m* [13.2*m*, 28.6*m*]	18.5*m* [12.0*m*; 27.3*m*]
**Median PFS, *months***	8.09*m* [4.2*m*, 13.3*m*]	7.9*m* [4.2*m*, 13.4*m*]
**Median PPS, *months***	5.8*m* [2.5*m*, 11.8*m*]	5.7*m* [2.5*m*, 11.6*m*]
	**N(%)**	**N(%)**
**Gender**		
Female	67 (64.4)	62 (66.0)
Male	37 (35.6)	32 (34.0)
**Ethnicity**		
Asian	41 (39.4)	36 (38.3)
Non-Asian	63 (60.6)	58 (61.7)
**Smoking history**		
Yes (ever-smokers)	46 (44.2)	41 (43.6)
No (never/non-smokers)	58 (55.8)	53 (56.4)
***EGFR* mutation type**		
Exon 19 deletion	46 (44.2)	42 (44.7)
Exon 21 (L858R)	39 (37.5)	36 (38.3)
‘All-other’ *EGFR*	19 (18.3)	16 (17.0)
**ECOG at TKI initiation**		
ECOG 0–1	51 (49.0)	44 (46.8)
ECOG 2–3	19 (18.3)	17 (18.1)
ECOG *Unknown*	34 (32.7)	33 (35.1)
**Family-lung cancer history**		
Yes	16 (15.4)	14 (14.9)
No	47 (45.2)	43 (45.7)
*Unknown*	41 (39.4)	37 (39.4)
**Comorbidities**		
Yes	54 (51.9)	51 (54.3)
No	49 (47.1)	42 (44.7)
NE/-or NA	1 (1.00)	1 (1.00)

ECOG was defined according to the Eastern Cooperative Oncology Group criteria [35]. Family-lung cancer history was defined as patients with an immediate-family member (mother/-or father/-or both; maternal/-or paternal aunt/-or uncle) with a positive history of lung cancer as reported by the attending oncologist in the patient’s medical history.

Comorbidities included the presence of hypertension and/-or diabetes (type I and/-or II) as reported by the attending oncologist in the patient’s medical history.

### Cohort (i) Stages IIIA-IV *EGFR*mut^+^ patients with RECIST-1.1-PD met (N = 104)

**Fig 1:**
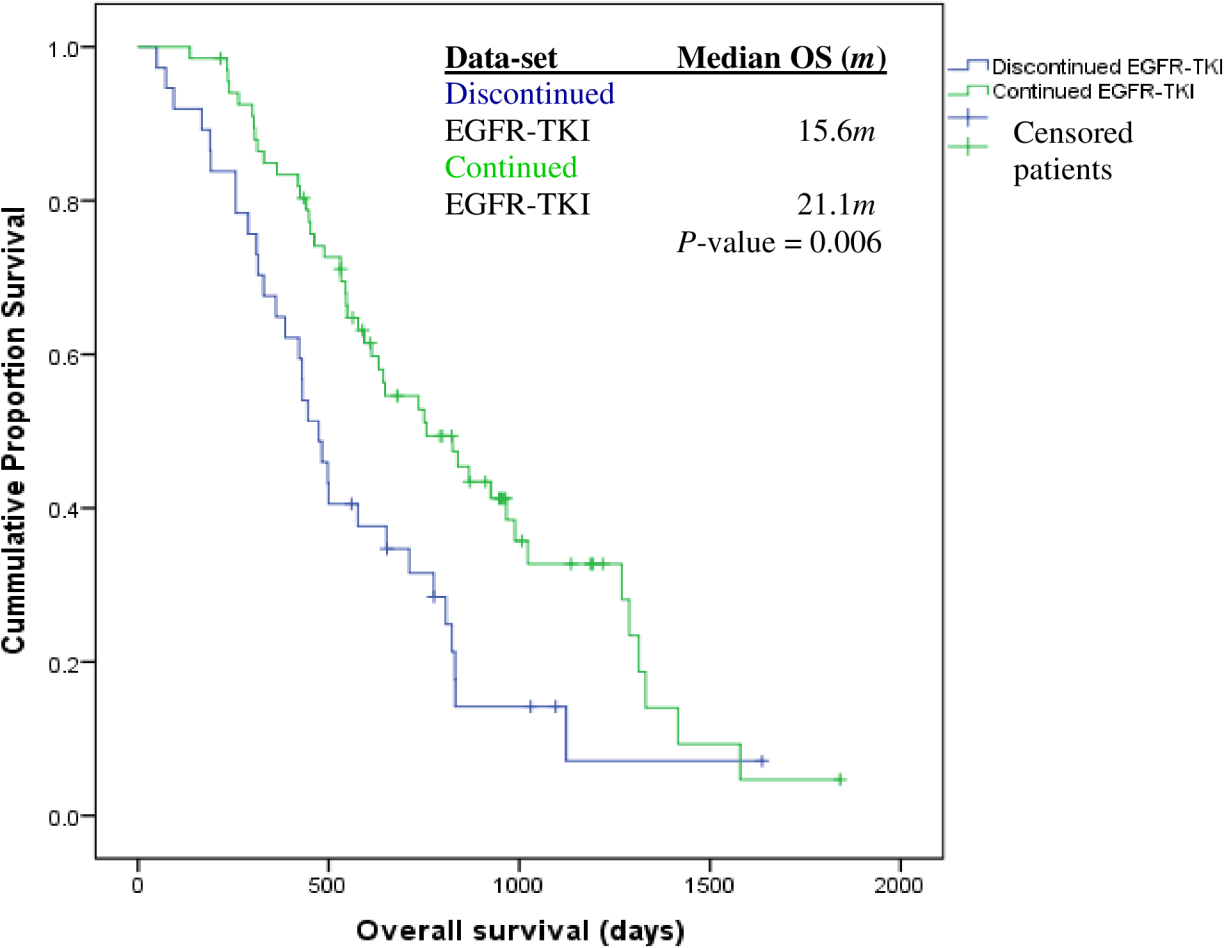
Overall survival of *EGFR*mut^+^ patients (stages IIIA-IV) who continued versus discontinued TKI treatment at initial-RECIST-1.1-PD (N = 67/104 vs. N = 37/104). **Continued** *EGFR*-TKI cohort (N = 45deaths/67); **Discontinued** *EGFR*-TKI cohort (N = 31 deaths/37). Overall survival was measured from diagnosis date to date of death/-or last follow-up date if still alive. Patients, who did not show or could not have been evaluated as per RECIST-1.1-PD criteria at the last study’s follow-up date, were censored on that date. The global log-rank test revealed a significant difference between the survival rates of each data-set, *P*-value = 0.006.

The median TKI treatment duration was 12.6 months (Q_1_: 6.04*m*; Q_3_: 18.5*m*). The median OS was 19.2 months (Q_1_: 13.0*m*; Q_3_: 28.6*m*), median PFS was 8.09 months; (Q_1_: 4.2*m*; Q_3_: 13.4*m*) and median PPS was 5.8 months (Q_1_: 2.4*m*; Q_3_: 11.9*m*). Patients who continued TKI treatment at initial-PD experienced longer OS, 21.1 months (Q_1_: 14.7*m*; Q_3_: 31.5*m*) versus those who discontinued it, 15.6 months (Q_1_: 9.9*m*; Q_3_: 25.5*m*), *p* = 0.006 (Fig 1). In addition, patients who continued TKI treatment were significantly older versus those who discontinued it; 68.5 years vs. 62.5 years, respectively, *P-*value < 0.001.

**Fig 2:**
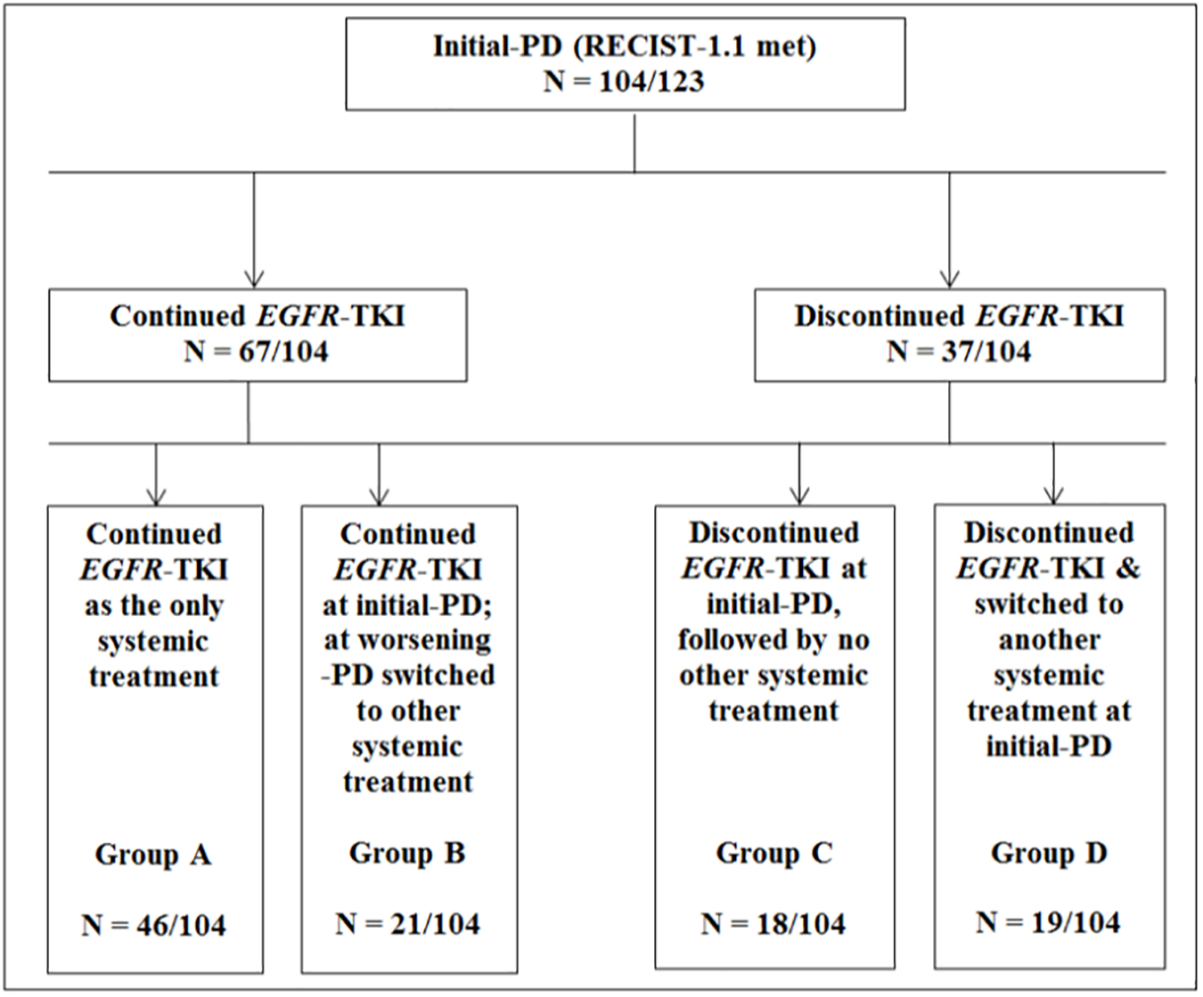
Treatment modality groups beyond initial disease-progression (N = 104–123). Other systemic treatments included one or more of the following: platinum-based chemotherapy: pemetrexed monotherapy, carboplatin + pemetrexed, cisplatin + pemetrexed, carboplatin + vinorelbine, cisplatin + vinorelbine, vinorelbine monotherapy, gemcitabine monotherapy, cisplatin + gemcitabine, carboplatin + gemcitabine, docetaxel, paclitaxel; clinical trials: IND.211, AURA 3, AZD9291; all others: Nivolumab.

**Fig 3:**
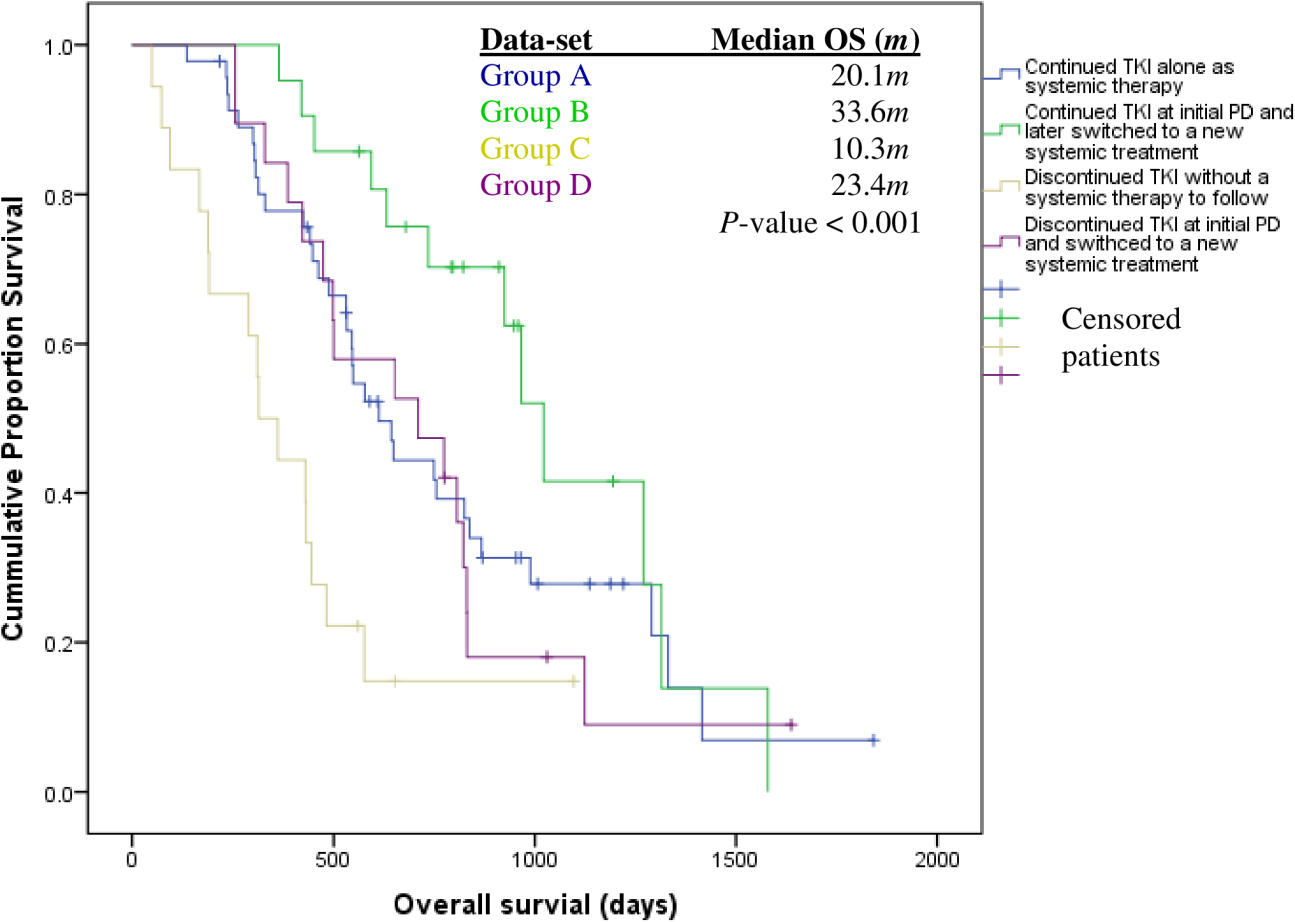
Overall survival of *EGFR*mut^+^ patients (stages IIIA-IV) according to post initial-RECIST-1.1-PD treatment pathways (N = 104/123). Continued ***EGFR*-TKI cohort: Group A (N = 33deaths/ 46); Group B (N = 12 deaths/ 21);** Discontinued ***EGFR*-TKI cohort: Group C (N = 15 deaths/18); Group D (N = 16 deaths/19). Group A**: *EGFR*mut^+^ NSCLC patients (IIIA-IV) who continued TKI treatment at initial-RECIST-1.1-PD, with no other lines of systemic therapy until death or last follow-up date. **Group B**: *EGFR*mut^+^ NSCLC patients (IIIA-IV) who continued TKI treatment at initial-RECIST-1.1-PD, followed by other systemic therapy until death or last follow-up date. **Group C**: *EGFR*mut^+^ NSCLC patients (IIIA-IV) who discontinued TKI treatment at initial-RECIST-1.1-PD, followed by no other line of systemic therapy until death or last follow-up date. **Group D**: *EGFR*mut^+^ NSCLC patients (IIIA-IV) who discontinued TKI treatment and were switched to a new line of systemic therapy at initial RECIST-1.1-PD. Statistical analysis (pairwise comparison using a False Discovery Rate (FDR) adjustment showed that (i) group C was different from all other treatment groups in terms of OS–versus Group A, adjusted log-rank test *P*-value = 0.008; versus Group B, adjusted log-rank test *P*-value = 0.0003; versus Group D, adjusted log-rank test *P*-value = 0.037; and that (ii) groups B and D were close to being statistically significantly different, with adjusted log-rank test, *P*-value = 0.078.

Treatment pathways from initial-PD onwards were identified, with the most common being TKI continuation, 64.4% (Fig 2). Other options included: continuing TKI alone—44.2% (46/104); continuing TKI + later switched to a new form of systemic therapy—20.2% (21/104); discontinuing TKI—17.3% (18/104), and lastly, discontinuing TKI to begin new systemic treatments—18.3% (19/104). Of note, immediate *EGFR*-TKI subsequent therapy included clinical trials with a monoclonal antibody and/-or platinum-based chemotherapy. Patients who continued treatment with a TKI at initial-PD and subsequently went on to receive new systemic treatment(s), experienced significant improved survival; 33.6 months, (Q_1_: 20.1*m*; Q_3_: 32.7*m*) versus patients who solely continued systemic treatment with TKI until death/or last follow-up date, 20.1 months, (Q_1_:13.2*m*; Q_3_:29.3*m*); *FDR-adjusted P-*value *= 0*.*078* (Fig 3).

From the MSM model, for the PD to death transition- smoking history, adjusted HR: 2.11 (95% CI: 1.13, 3.92), and all post-PD treatments were independent prognostic factors for OS after adjusting for clinicopathological variables between diagnosis and PD, as well as from PD to death, from any cause (S1 Table). Patients that continued TKI at initial-PD and were later switched to a new systemic therapy, experienced an 83% reduced risk of death, adjusted HR: 0.17 (95% CI: 0.07, 0.39), meanwhile patients who switched from a TKI to a new systemic treatment at initial-PD, experienced only a 61% reduced risk of death, adjusted HR: 0.39 (95% CI: 0.18, 0.84). The weakly significant interaction between gender and smoking history revealed an increased risk of death in female ever-smokers, adjusted HR: 2.11 (95% CI: 1.14, 3.92, Table 2).

**Table 2:** From MSM model: Assessing two-way interactions between gender and smoking history for post-progression survival (N = 104/123).

	Smoking History	
Gender	No (Never/Non-Smoker)	Yes (Ex/-or Current Smoker)
Female (baseline)	HR = 1	HR = 2.11 [95% CI = 1.14, 3.92]
Male	HR = 1.82 [95% CI = 0.90, 3.70]	HR = 1.49 [95% CI = 0.77, 2.90]

### Cohort (ii) PPS for stage IV *EGFR*mut^+^ patients (N = 94)

Significant PPS differences were found between (i) patients who continued TKI at initial-PD and subsequently were switched to platinum-based chemotherapy, adjusted HR: 0.15 (95% CI: 0.08,0.30), as well as for (ii) those who switched from a TKI to a new systemic therapy at initial-PD, adjusted HR: 0.29 (95% CI: 0.15, 0.54) (S2 Table).

Interactions between smoking history and *EGFR* mutation type (exon 19, exon 21 and ‘all other’ *EGFR* mutations) were identified in post-PD survival analysis, *P-*value = 0.113 for exon 21(L858R) and *p* = 0.104 for ‘all other’ *EGFR* mutations, signifying a weak non-independent relationship between *EGFR* mutation type and smoking history. Across all groups (Table 3), the ever-smoker patients with an exon 19 deletion, had more than three times higher risk of post-PD death (adjusted HR: 3.19; 95% CI:1.54, 6.58), and those carrying an exon 21 mutation, with a smoking history, had more than double the risk (adjusted HR: 2.10; 95% CI:1.10, 4.00). Further, ever-smoker patients, carrying ‘all other’ *EGFR* mutation had similar non-significant risks of death as patients with a negative smoking history, regardless of their *EGFR* mutation type. As outlined in Table 3, ever-smoker females experienced significant elevated risks of post-PD death (HR: 3.19; 95% CI: 1.54, 6.58), compared to non-smokers of either gender. Of further note, male ever-smokers had a 93% increase in risk of post-PD death, although the 95% confidence interval included the null value of 1 (95% CI: 0.94, 3.99). Similar results were found in the MSM model for the gender–smoking history interaction from PD to death.

**Table 3:** From Cox PH with propensity score weights model for Stage IV patients only: Assessing two-way interactions between gender and smoking history and *EGFR* mutation type and smoking history for post-progression survival (N = 94/104).

	Smoking History	
Gender	No (Never/Non-Smoker)	Yes (Ex/-or Current Smoker)
Female (baseline)	HR = 1	HR = 3.19 [95% CI = 1.54, 6.58]
Male	HR = 1.39 [95% CI = 0.82, 2.35]	HR = 1.49 [95% CI = 0.77, 2.90]
***EGFR* mutation type**		
Ex 19 deletion (baseline)	HR = 1	HR = 3.19 [95% CI = 1.54, 6.58]
Ex 21 (L858R)	HR = 1.24 [95% CI = 0.70, 2.20]	HR = 2.10 [95% CI = 1.10, 4.00]
‘All-other’ *EGFR*	HR = 1.30 [95% CI = 0.44, 3.86]	HR = 1.18 [95% CI = 0.43, 3.25]

Cox PH Model adjusted for these variables: Variables included sex, smoking status, *EGFR* mutation type, post- progression treatments (continued TKI alone, continued TKI plus other systemic therapy, discontinued TKI plus systemic therapy, and no systemic therapy), interactions between sex and smoking status and *EGFR* mutation type and smoking status.

## Discussion

Small-molecule *EGFR*-TKIs have provided significant improved outcomes for patients harbouring sensitizing *EGFR* mutations. The management of these patients’ post-TKI-progression represents a new therapeutic challenge. Nonetheless, the criteria of clinical benefit associated with continuing TKIs beyond RECIST-1.1-defined-PD remains poorly defined due to the lack of investigation with this approach [26]. Thus, the greatly debated issue of whether or not to continue TKI treatment beyond PD was analyzed in this study by investigating the variance in treatment pathways at initial-PD and associated outcomes. As 64% of patients continued TKI treatment beyond initial PD, this indicated a common practice at our centre. Of note, the median PFS of 8.09 months was consistent with other similar studies, where PFS ranged from approximately 8–11 months [27]. In addition, the median PPS (5.8 months) was also comparable with other clinical studies, where median PPS after TKI-PD ranged between 4.0–12.0 months [28–30].

The IMPRESS trial evaluated the impact of continuing TKI treatment (gefitinib) in addition to chemotherapy concurrently after initial-TKI progression versus chemotherapy alone on PFS. IMPRESS demonstrated that there is no value in continuing the *EGFR*-TKI at the time of disease-progression on PFS [21]. Our study however, assessed the clinical outcomes associated with maintaining patients on a TKI-monotherapy at initial-PD and later switching to a new systemic treatment. Our findings highlight favourable outcomes in PPS if *EGFR*mut^+^ NSCLC patients carry TKI treatment beyond initial-PD especially in combination with a new systemic therapy. Further, our study confirms the benefits found by the ASPIRATION trial, where continuing *EGFR*-TKI treatment beyond PD was valuable. Particularly, our study showed that amongst both sub-groups; (i) stage IIIA-IV patients and (ii) stage IV *EGFR*mut^+^ NSCLC patients, continuing TKI treatment at initial-PD, provided a PPS advantage versus discontinuing the TKI, even when other systemic therapy was provided. Additionally, although the ASPIRATION trial focused on post TKI-PD management, there was a lack of control arm, which would have allowed measuring the separate effect of continuing erlotinib beyond initial-TKI-PD. Our results showed that continuing TKI treatment beyond initial-progression as the only means of systemic treatment until death or last follow-up date, prolonged post-PD survival better in comparison to discontinuing the TKI at initial-PD with no other subsequent systemic treatments. Further, by continuing TKI at initial-PD, patients could potentially gain time needed to provide a new tissue biopsy for identification of resistant-TKI mutations, such as the T790M. This could therefore narrow the gap between the re-biopsy result and initial PD-management, and guide oncologists to less toxic and effective subsequent-PD treatment options, such as the recently approved, third-generation *EGFR*-TKI, osimertinib.

Significant interactions between smoking history, gender and *EGFR* mutation type were also identified when OS and PPS were analyzed, after adjusting for baseline characteristics whether they were statistically significant or not. Specifically, female ever-smokers had the worst risk of death for PPS, although male ever-smokers also experienced an increased, yet not statistically significant, risk of death. Despite other studies outlining OS differences according to *EGFR* mutation type, smoking history and ethnicity [4,31,32], our study is the first to identify and describe a non-independent relationship between the type of *EGFR* mutation and smoking history within a mixed-ethnicity *EGFR*mut^+^ cohort. In particular, smoking status upstaged the impact on PPS in comparison to the *EGFR* mutation type and ethnicity; ever-smokers with an exon 19 deletion experienced approximately three times higher a risk of death, post-PD, even after adjusting for clinicopathological variables, meanwhile ever-smokers and with an exon 21 mutation, had more than double the risk of death after initial-PD compared to non-smokers or smokers harbouring an ‘all other’ *EGFR* mutation. Thus, amongst *EGFR*mut^+^ NSCLC patients at our centre, the risk of post-PD death may be accelerated or slowed down by the combined effects of smoking history and type of *EGFR* mutation, with the possibility of a synergistic relationship.

The retrospective nature of this non-randomized study does have intrinsic limitations. Overlaps between different lines of systemic therapy post initial-PD may have occurred- influencing both OS and PPS, and choosing to continue with a TKI treatment was not a randomized strategy, therefore knowing its true effect on OS remains somewhat limited. For example, if an oncologist continues treatment at progression with a TKI in a patient with poor performance status, it is more likely for that patient to have a poorer prognosis overall, than a patient who might be younger and have good functional status in order to be switched to a platinum-based regimen. Thus, PD-treatment heterogeneity and the lack of following randomized practice at the time of PD in *EGFR*mut^+^ patients make post-progression treatment strategies speculative on the basis of this study. Furthermore, the different time intervals for assessing progression after initial-TKI treatment could have also influenced subsequent treatment.

Regression models used to analyze the survival in this study allowed optimal adjustment of multiple explanatory variables (i.e., previous TKI lung-cancer related treatment(s), gender, smoking history, ethnicity, *EGFR* mutation type, and post TKI systemic treatments), and minimized bias in this observational study. By creating more homogeneous cohorts of patients for each set of analysis, propensity score weights and MSM maintained statistical power compared to the evaluation of multiple patient subgroups. Other studies have also investigated subsequent treatments beyond TKI-PD, however modeling of survival was mostly carried out via Kaplan-Meier curves [28–30,33,34], which does not account for factors that could impact survival and/-or censoring times.

Overall, our study suggests that continuing TKI as a monotherapy at initial-PD and later switching to other systemic treatment, provides a survival gain versus discontinuing the TKI, and that the interactions between smoking status and gender or *EGFR* mutation type, were highly suggestive that the effect of smoking has a greater impact on PPS than gender and *EGFR* mutation type alone.

## Supporting information

S1 Fig Consort diagram of patient selection. (DOCX)

S2 Fig Transition states for MSM used to estimate OS (top) and Cox PH regression model with propensity score weights used to estimate PPS (bottom). (DOCX)

S3 Fig Index plots of *dfbetas* for the Cox PH regression analysis of PPS on gender, smoking history, *EGFR* mutation type and post-PD treatments. (DOCX)

S4 Fig Effect size plot before and after weighting of predictors of TKI continuation or discontinuation at initial progression (Top: closed red circles indicate a statistically significant difference) and (Lower: Q-Q-plot of ordered *t-*test *P*-values versus quantiles of the uniform distribution for individual predictors before and after weighting). (DOCX)

S1 Table MSM (separate PHs assumed for transitions 1 and 2, with adjustment for clinicopathological variables between diagnosis to initial-PD) (N = 104 transition 1; N = 76 transition 2). (DOCX)

S2 Table From Cox PH with propensity score weights in Stage IV patients (N = 94). (DOCX)
