# Supplementary material for: Beyond disease-progression: Clinical outcomes after EGFR-TKIs in a cohort of EGFR mutated NSCLC patients
Source: PLoS One. 2017 Aug 4;12(8):e0181867. doi: 10.1371/journal.pone.0181867 (PMC5544231; doi:10.1371/journal.pone.0181867)
Supplement: S1 Fig — (DOCX) [file pone.0181867.s001.docx]

# Supplemental Figure

### S1 Fig. Consort diagram of patient selection.

Alberta Cancer

Registry

Calgary Lab Services

IRESSA Alliance Program

Total NSCLC reviewed

(2010-2014)

N = 2,652

Glans-Look

Lung Cancer

Database

Confirmed *EGFR*mut^+^ NSCLC treated with *EGFR*-TKI

N = 140/2,652

**N = 2,652**

Electronic Medical Record

No access to patient records

N = 2/140

Final *EGFR*mut^+^ NSCLC cohort

N = 138/140

PD-met *de-novo* stages IIIA-IV *EGFR*mut^+^ NSCLC patients

N = 104/123

RECIST – 1.1 PD criteria [1] not met

(N = 19/138) or *de-novo* Stage < III (N=15/138)

PD-met de-novo stage IV *EGFR*mut^+^ NSCLC patients

N = 94/104

1. Oken MM, Creech RH, Tormey DC, Horton J, Davis TE, McFadden ET, et al: Toxicity and response criteria of the Eastern Cooperative Oncology Group. Am J Clin Oncol 5:649-655, 1982
