# Supplementary material for: Beyond disease-progression: Clinical outcomes after EGFR-TKIs in a cohort of EGFR mutated NSCLC patients
Source: PLoS One. 2017 Aug 4;12(8):e0181867. doi: 10.1371/journal.pone.0181867 (PMC5544231; doi:10.1371/journal.pone.0181867)
Supplement: S2 Fig — (DOCX) [file pone.0181867.s002.docx]

# Supplemental Figure

### S2 Fig. Transition states for MSM used to estimate OS (top) and Cox PH regression model with propensity score weights used to estimate PPS (bottom).

Top: MSM; *de novo* Stage IIIA-IV, N= 123

Lower: Cox PH model with propensity score weights; *de novo* Stage IV meeting RECIST -1.1-PD criteria, N = 94

**N= 104**

DIAGNOSIS

N = 11/123 ^no PD^

PD (RECIST-1.1)

N = 28/104 ^no death^

1

**N= 76**

2

**N= 8**

3

DEATH

STAGE IVwithRECIST-1.1-PD

**N= 94**

DEATH

4

**S2 Fig legend**. **Top (MSM; *de novo* Stage IIIA-IV, N= 123):** Transition from diagnosis to disease-progression according to RECIST-1.1 criteria labeled as #1; transition from diagnosis to direct death labeled as #3; and transition from disease-progression to death labeled as #2. Cox PH regression models were adopted for all transition intensities that did not assume they were proportional to each other. **Transition 1** for patients with de-novo stage IIIA-IV disease adjusted for baseline patient, tumour and treatment variables (sex, smoking status, ethnicity, mutation type, age at diagnosis, family history of lung cancer, radiation therapy prior to TKI initiation, chemotherapy prior to TKI initiation, surgery prior to TKI initiation, pleural effusion present at TKI initiation, other treatment prior to TKI initiation) between diagnosis to initial-PD. **Transition 2** (PD to death) adjusted for sex, smoking status, initial stage (IIIA, IIIB or IV) and stage at TKI initiation (IIIA, IIIB or IV) and post-progression treatments (continued TKI alone, continued TKI plus other systemic therapy, discontinued TKI plus systemic therapy, and no systemic therapy). **Lower (Cox PH model with propensity score weights; *de novo* Stage IV meeting RECIST -1.1-PD criteria, N = 94):** Average treatment effect (ATE) approach was used to evaluate the probability to continue or not with a TKI at progression. Propensity score weights were estimated from a logistic regression model that included initial age at diagnosis, family history of lung cancer, progression-free survival interval (initial PD – TKI initiation), radiation therapy prior to TKI initiation, pleural effusion present at TKI initiation plus other baseline characteristics that had no relative influence (radiation or chemotherapy or surgery prior to *EGFR*-TKI start for initial diagnosis, pathological histology at diagnosis, initial stage, stage at TKI initiation, radiation or chemotherapy or other treatments prior *EGFR*-TKI initiation and *EGFR* mutation type). **Transition 4** (PD to death) was estimated with a Cox PH regression model using the propensity score weights and robust standard initiation and *EGFR* mutation type). **Transition 4** (PD to death) was estimated with a Cox PH regression model using the propensity score weights and robust standard errors. Variables included sex, smoking status, *EGFR* mutation type, post-progression treatments (continued TKI alone, continued TKI, followed by a switch to a new systemic therapy at subsequent worsening-PD, discontinued TKI and switched to a new form of systemic therapy, and no systemic therapy), interactions between sex and smoking status and *EGFR* mutation type and smoking status.
