# Supplementary material for: Beyond disease-progression: Clinical outcomes after EGFR-TKIs in a cohort of EGFR mutated NSCLC patients
Source: PLoS One. 2017 Aug 4;12(8):e0181867. doi: 10.1371/journal.pone.0181867 (PMC5544231; doi:10.1371/journal.pone.0181867)
Supplement: S3 Fig — (DOCX) [file pone.0181867.s003.docx]

### Supplemental Figure

### S3 Fig. Index plots of *dfbetas* for the Cox PH regression analysis of PPS on gender, smoking history, *EGFR* mutation type and post-PD treatments.


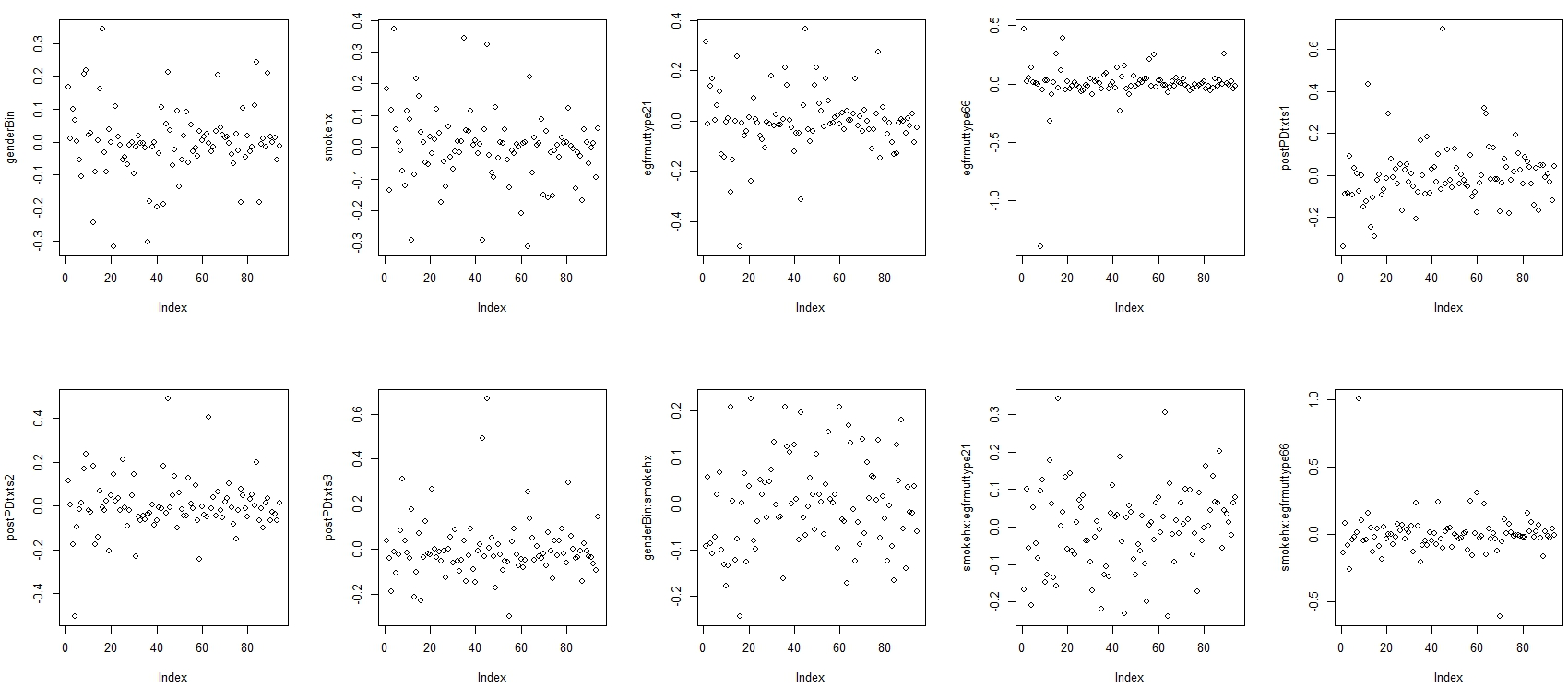


^2^**S3 Fig legend.** Influential patients (outliers) that could have shifted outcomes were also checked. Although a few patients did not fit the final Cox PH model and had *dfbetas* larger than other patients, this did not alter overall survival and PPS conclusions made in the results section of this manuscript, when they were removed from the analysis.
