# Supplementary material for: Beyond disease-progression: Clinical outcomes after EGFR-TKIs in a cohort of EGFR mutated NSCLC patients
Source: PLoS One. 2017 Aug 4;12(8):e0181867. doi: 10.1371/journal.pone.0181867 (PMC5544231; doi:10.1371/journal.pone.0181867)
Supplement: S4 Fig — (DOCX) [file pone.0181867.s004.docx]

### Supplemental Figure

### S4 Fig. Effect size plot before and after weighting of predictors of TKI continuation or discontinuation at initial progression (Top: closed red circles indicate a statistically significant difference) and (Lower: Q-Q-plot of ordered *t­-*test *P*-values versus quantiles of the uniform distribution for individual predictors before and after weighting).


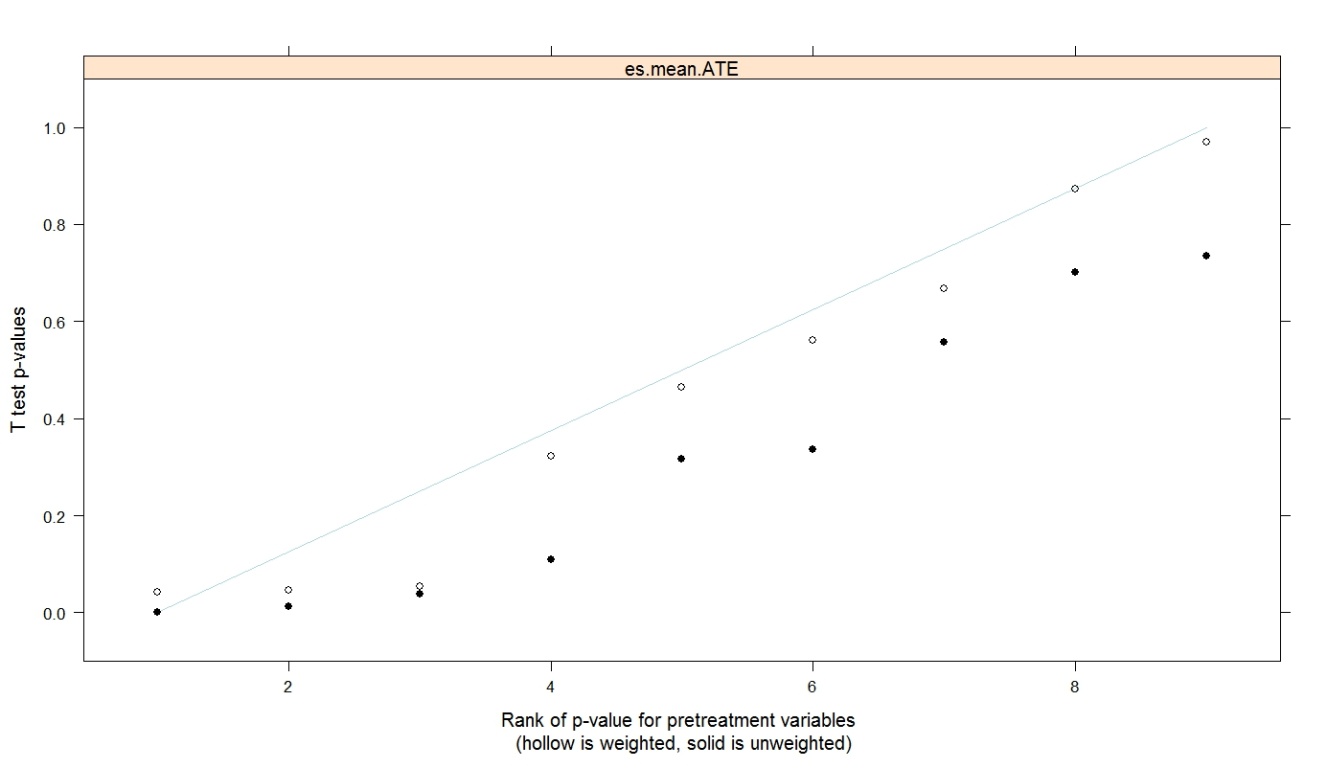
**
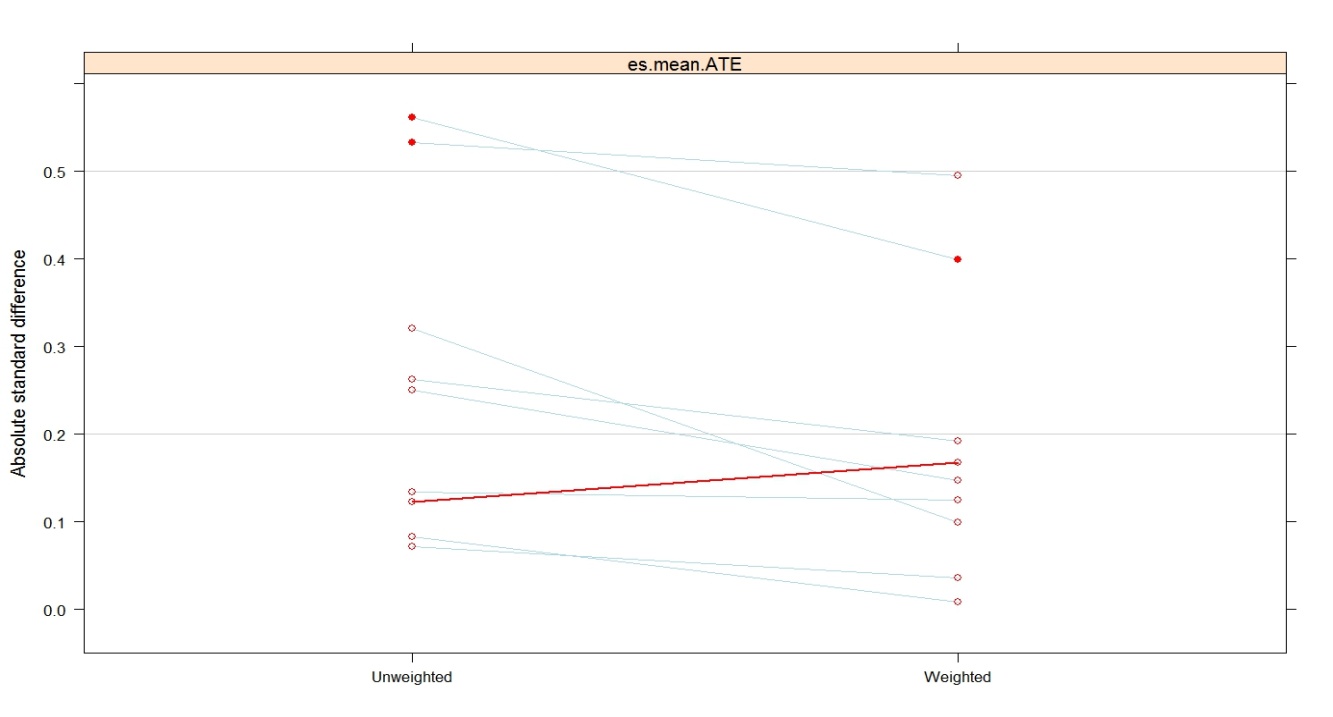
**
