# Supplementary material for: Beyond disease-progression: Clinical outcomes after EGFR-TKIs in a cohort of EGFR mutated NSCLC patients
Source: PLoS One. 2017 Aug 4;12(8):e0181867. doi: 10.1371/journal.pone.0181867 (PMC5544231; doi:10.1371/journal.pone.0181867)
Supplement: S1 Table — (DOCX) [file pone.0181867.s005.docx]

# Supplemental Table

### S1 Table. MSM (separate PHs assumed for transitions 1 and2, with adjustment for clinicopathological variables between diagnosis to initial-PD) (N= 104 transition 1; N= 76 transition 2).

|  | **HR** | **95%CI (Lower, Upper)** | ***P*-value** |
| --- | --- | --- | --- |
| ***Transition 1: From diagnosis to time of developing RECIST-1.1-PD (N=123)*** | | | |
| **Patient variables** |  | | |
| Gender (F=baseline) | 1.04 | 0.67, 1.62 | 0.868 |
| Age at diagnosis | 0.97 | 0.95, 0.99 | 0.001^*^ |
| Smoking history (Never/non-smoker=baseline) | 1.25 | 0.79, 1.98 | 0.348 |
| Ethnicity(Asian=baseline) | 0.77 | 0.47, 1.27 | 0.307 |
| Family lung-cancer history | 2.45 | 1.31, 4.56 | 0.005^*^ |
| Initial stage (stage IV=baseline) |  | | |
| IIIA | 0.29 | 0.04, 2.23 | 0.235 |
| IIIB | 0.11 | 0.02, 0.67 | 0.017* |
| TKI-start stage (stage IV=baseline) |  | | |
| IIIA | 0.31 | 0.05, 1.86 | 0.200 |
| IIIB | 6.74 | 0.68, 66.91** | 0.103 |
| Tumor variables (Ex 19del=baseline) |  | | |
| Ex 21 (L858R) | 1.35 | 0.82, 2.21 | 0.240 |
| ‘All-other’ *EGFR* | 1.27 | 0.67, 2.41 | 0.472 |
| Pleural Effusion | 0.83 | 0.49, 1.39 | 0.468 |
| Treatment variables |  | | |
| Radiation prior TKI | 1.90 | 1.13, 3.19 | 0.014^*^ |
| Platinum-based chemotherapy prior TKI | 0.66 | 0.21, 2.06 | 0.479 |
| Surgery prior TKI | 1.84 | 0.26, 12.80^**^ | 0.540 |
| ***Transition 2: From RECIST-1.1-PD to death/-or last follow-up date (N=104/123)*** | | | |
| **Patient variables** |  | | |
| Gender(F=baseline) | 1.82 | 0.90, 3.70 | 0.096 |
| Smoking history(Never/non-smoker=baseline) | 2.11 | 1.13, 3.92 | 0.018^*^ |
| Gender*Smoking history | 0.39 | 0.14, 1.05 | 0.061 |
| **Treatment variables**  (Post-PD treatments-4=baseline) |  | | |
| Post-PD treatments-1 | 0.53 | 0.27, 1.01 | 0.055 |
| Post-PD treatments-2 | 0.17 | 0.07,0.39 | < 0.00001 |
| Post-PD treatments-3 | 0.39 | 0.18, 0.84 | 0.016^*^ |

**S1 table legend.** The baseline *EGFR* mutation was exon 19 deletion, meanwhile the baseline for post-PD treatment pathway was discontinue *EGFR*-TKI treatment at initial-PD (post-PD treatments-4 sub-group), followed by no other lines of systemic treatment. Stage IV was used as the baseline for initial stage and TKI-start. **Post-PD treatments -1**: *EGFR*mut^+^ NSCLC patients (de-novo stage IV only) who continued TKI treatment at initial-RECIST-1.1-PD, followed by no other systemic line of therapy until death or last follow-up date. **Post-PD treatments -2**: *EGFR*mut^+^ NSCLC patients (de-novo stage IV only) who continued TKI treatment at initial-RECIST-1.1-PD, and at subsequent worsening-PD were switched to a new form of systemic therapy until death or last follow-up date. **Post-PD treatments -3**: *EGFR*mut^+^ NSCLC patients (de-novo stage IV only) who discontinued TKI treatment at initial-RECIST-1.1-PD, and were switched to a new line of systemic therapy.

**P*-values represent significant statistical difference was met.

**95% Confidence Intervals signified wide confidence intervals. 95% Confidence Intervals were rounded up to 2 decimal places, where applicable.
