# Supplementary material for: Beyond disease-progression: Clinical outcomes after EGFR-TKIs in a cohort of EGFR mutated NSCLC patients
Source: PLoS One. 2017 Aug 4;12(8):e0181867. doi: 10.1371/journal.pone.0181867 (PMC5544231; doi:10.1371/journal.pone.0181867)
Supplement: S2 Table — (DOCX) [file pone.0181867.s006.docx]

# Supplemental Table

### S2 Table. From Cox PH with propensity score weights in Stage IV patients (N= 94).

|  | **HR** | **95%CI (Lower, Upper)** | | ***P*-value** |
| --- | --- | --- | --- | --- |
| **Patient variables** |  | | | |
| Gender (F=baseline) | 1.39 | | 0.84, 2.35 | 0.217 |
| Smoking history (Never/non-smoker=baseline) | 3.19 | | 1.54, 6.58 | 0.0017^*^ |
| Gender (F=baseline)*  Smoking history (Never/non-smoker=baseline)  [***interaction***] | 0.44 | | 0.22, 0.88 | 0.021^*^ |
| **Tumor variables** (Ex 19 del=baseline) |  | | | |
| Ex 21 (L858R) | 1.24 | | 0.70, 2.20 | 0.466 |
| ‘All other’ *EGFR* | 1.30 | | 0.44, 3.86 | 0.634 |
| Smoking history*  Exon 21 (L858R)  [***interaction***] | 0.53 | | 0.24, 1.16 | 0.113 |
| Smoking history*  ‘All-other’ mutation  [***interaction***] | 0.28 | | 0.06, 1.0 | 0.104 |
| **Treatment variables**  (Post-PD treatments-4=baseline) |  | | | |
| Post-PD treatments -1 | 0.58 | | 0.31, 1.08 | 0.088 |
| Post-PD treatments -2 | 0.15 | | 0.08, 0.30 | < 0.00001^*^ |
| Post-PD treatments -3 | 0.29 | | 0.15, 0.54 | < 0.0001^*^ |

**S2 table legend.** The baseline *EGFR* mutation was exon 19 deletion, meanwhile the baseline for post-PD treatment pathway was discontinue *EGFR*-TKI treatment at initial-PD (post-PD treatments-4 sub-group), followed by no other lines of systemic treatment. Stage IV was used as the baseline for initial stage and TKI-start. **Post-PD treatments-1**: *EGFR*mut^+^ NSCLC patients (de-novo stage IV only) who continued TKI treatment at initial-RECIST-1.1-PD, followed by no other systemic lines of therapy until death or last follow-up date. **Post-PD treatments-2**: *EGFR*mut^+^ NSCLC patients (de-novo stage IV only) who continued TKI treatment at initial-RECIST-1.1-PD, and at subsequent worsening-PD were switched to a new form of systemic therapy until death or last follow-up date. **Post-PD treatments-3***: EGFR*mut^+^ NSCLC patients (de-novo stage IV only) who discontinued TKI treatment at initial-RECIST-1.1-PD, and were switched to a new line of systemic therapy.

**P*-values represent significant statistical difference was met.

**95% Confidence Intervals were rounded up to 2 decimal places, where applicable.
